# FZD5 contributes to TNBC proliferation, DNA damage repair and stemness

**DOI:** 10.1038/s41419-020-03282-3

**Published:** 2020-12-12

**Authors:** Yu Sun, Zhuo Wang, Lei Na, Dan Dong, Wei Wang, Chenghai Zhao

**Affiliations:** Department of Pathophysiology, College of Basic Medical Science, China Medical University, Shenyang, China

**Keywords:** Breast cancer, DNA damage and repair

## Abstract

Chemotherapy currently remains the standard treatment for triple-negative breast cancer (TNBC). However, TNBC frequently develop chemoresistance, which is responsible for cancer recurrence and distal metastasis. Both DNA damage repair and stemness are related to chemoresistance. FZD5, a member in Frizzled family, was identified to be preferentially expressed in TNBC, and associated with unfavorable prognosis. Loss and gain of function studies revealed that FZD5 contributed to TNBC cell G1/S transition, DNA replication, DNA damage repair, survival, and stemness. Mechanistically, transcription factor FOXM1, which promoted BRCA1 and BIRC5 transcription, acted as a downstream effecter of FZD5 signaling. FOXM1 overexpression in FZD5-deficient/low TNBC cells induced FZD5-associated phenotype. Finally, Wnt7B, a specific ligand for FZD5, was shown to be involved in cell proliferation, DNA damage repair, and stemness. Taken together, FZD5 is a novel target for the development of therapeutic strategies to overcome chemoresistance and prevent recurrence in TNBC.

## Introduction

Breast cancer is the most common malignant tumor in women. Early detection and systemic therapies have decreased the mortality in North America and the European Union^1^. Triple-negative breast cancer (TNBC) currently lack established molecular targets, and TNBC patients usually have an unfavorable prognosis. Cytotoxic chemotherapy remains the standard treatment for TNBC^2^. Although TNBC generally have a high response rate to chemotherapy, they frequently develop chemoresistance^3^. Therefore, it is crucial to identify new molecular targets and develop novel strategies by elucidating the mechanisms of chemoresistance.

In response to endogenous and exogenous insults, normal cells utilize a variety of DNA damage sensing and repair mechanisms, also called DNA damage response (DDR), to maintain genomic integrity. Compared to normal cells, malignant cells have a higher DNA damage burden because of oncogene-induced replication stress^4^. Accordingly, malignant cells usually have enhanced DNA repair capacity to cope with DNA damage and to proliferate and survival. The increase in DNA repair capacity weakens the therapeutic effect of radiotherapy and chemotherapy, which induce cell death through DNA damage, thereby causing radioresistance and chemoresistance, and cancer recurrence. In TNBC, several factors such as tripartite motif containing 37 (TRIM37), neuropilin 2 (NRP2), and dynamin 2 (DNM2) have recently been shown to be associated with DNA damage repair, contributing to chemoresistance^5–7^.

It has been widely accepted that a subpopulation of cells within tumors have stem-like properties (stemness). These cells are also named cancer stem cells (CSC), responsible for tumorigenesis, therapy resistance and relapse. The ability of CSC to survive chemotherapy and radiotherapy correlates with prompt activation of DDR^8,9^. In TNBC, CSC display heterogeneity, with different markers, such as CD44/CD24, ALDH1, CD133, and EPCAM^10–14^. Stemness endows TNBC cells with metastatic potential^15–17^ and chemoresistance^18,19^. Notably, chemotherapy can increase CSC population in TNBC cell lines or primary tumors^20,21^. Targeting CSC has been shown to sensitize TNBC cells to chemotherapy^22–24^.

Here Frizzled 5 (FZD5), a member in FZD family, was identified as a factor promoting DNA damage repair, stemness, and chemoresistance in TNBC cells. Moreover, transcription factor Forkhead Box M1 (FOXM1), which modulated BRCA1 and BIRC5 (Survivin) transcription, was demonstrated to be a downstream effector of FZD5 signaling.

## Results

### FZD5 is principally expressed in TNBC, and associated with unfavorable prognosis

**Fig. 1:**
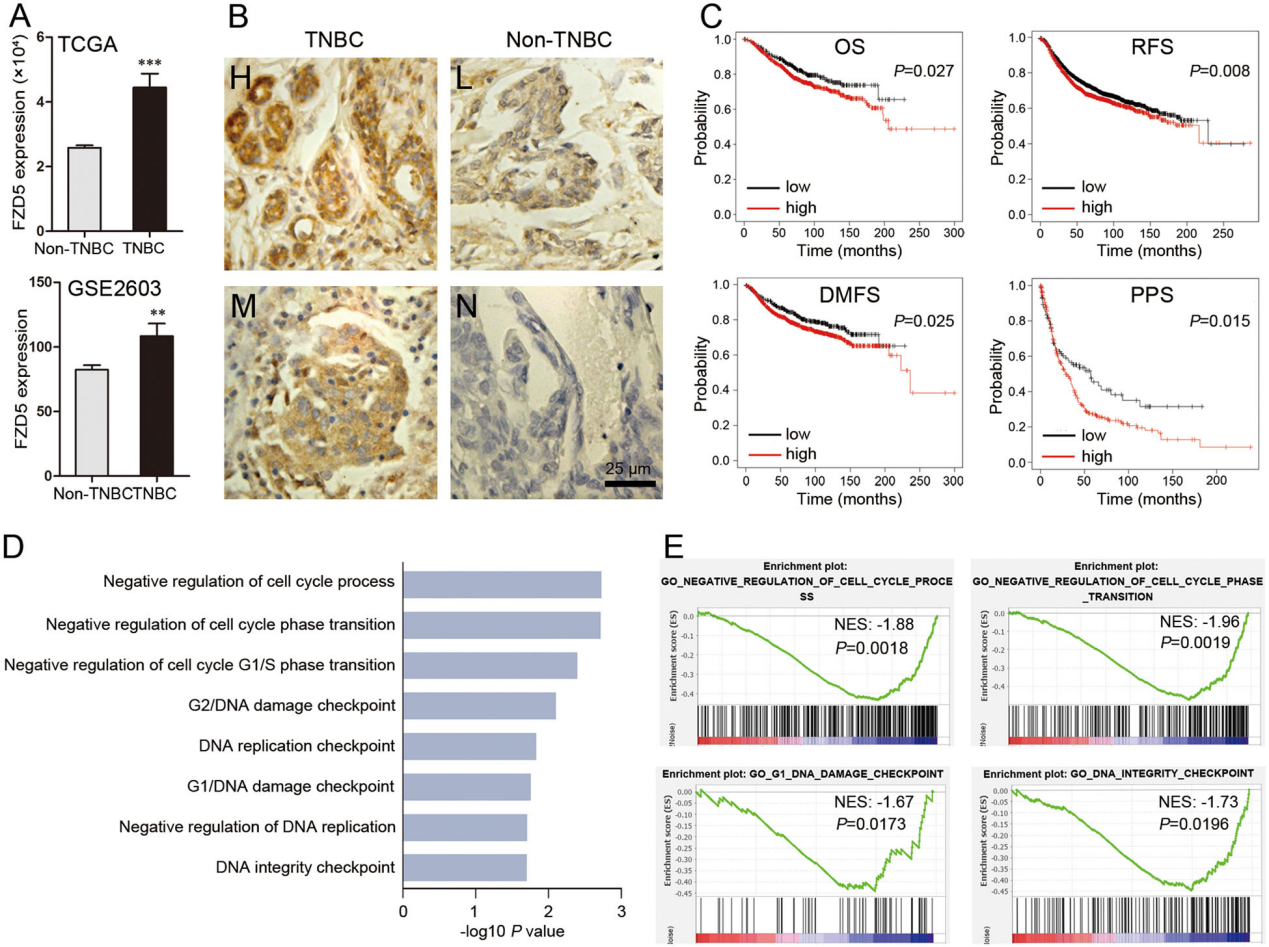
FZD5 is principally expressed in TNBC and associated with unfavorable prognosis. **A** TCGA (TNBC: 115; non-TNBC: 858) and GSE2603 (TNBC: 25; non-TNBC: 71) databases were interrogated for FZD5 expression. FZD5 mRNA levels were compared between TNBC and non-TNBC. **P < 0.01, ***P < 0.001, vs. non-TNBC. **B** FZD5 expression in 18 TNBC and 24 non-TNBC was detected by immunohistochemistry. Staining of some representative samples was shown. H: high; M: median; L: low; N: negative. Scalebar: 25 μm. **C** The association of FZD5 with OS, RFS, DMFS, and PPS was analyzed in Kaplan–Meier plotter website. **D**, **E** Go analysis of the gene pathways differentially expressed between FZD5-high and FZD5-low breast cancer samples in TCGA database was performed. Four representative GSEA-enrichment plots were shown.

The Cancer Genome Atlas (TCGA) database was interrogated for FZD5 mRNA expression in breast cancer tissues. TNBC expressed a higher level of FZD5 mRNA compared to non-TNBC (Fig. 1A). This finding was supported by the interrogation of GSE2603 database (Fig. 1A). The preferential expression of FZD5 in TNBC was further confirmed by Immunohistochemistry staining (Fig. 1B). 15 of 18 TNBC presented positive staining, while only 4 of 24 non-TNBC appeared FZD5-positive (*χ*^2^ = 18.45, *P* < 0.0001). The association of FZD5 with survival was analyzed in Kaplan–Meier plotter website. Higher FZD5 expression was correlated with shorter overall survival (OS), recurrence-free survival (RFS), distal metastasis-free survival (DMFS), and post-progression survival (PPS) (Fig. 1C). Gene set enrichment analysis (GSEA) of TCGA database suggested that FZD5 was related to cell cycle transition, DNA replication, and DNA damage repair (Fig. 1D, E). GSEA further indicated that FZD5 was implicated in a series of signaling pathways related to chemo-resistance or radio-resistance (Supplementary Table 1, Supplementary Fig. 1).

### FZD5 induces cell growth in vitro and in vivo

**Fig. 2:**
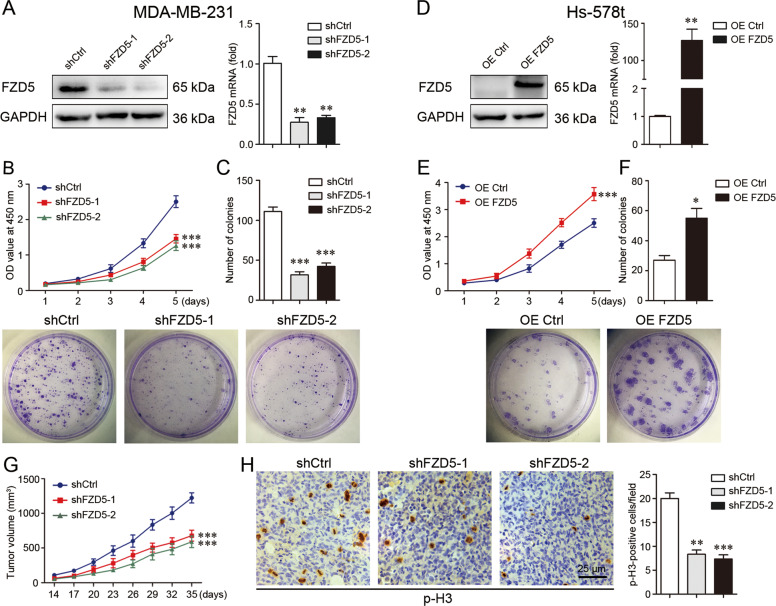
FZD5 induces cell growth in vitro and in vivo. **A** FZD5 expression in MDA-MB-231 cells stably transfected with shCtrl or shFZD5-1/2 was detected by Western blot and real-time PCR. **B** Viability of MDA-MB-231 cells was analyzed by CCK8. **C** Growth of MDA-MB-231 cells was determined by Colony formation, mean ± SD, *n* = 3. **P < 0.01, ***P < 0.001, vs. shCtrl. **D** FZD5 expression in Hs-578t cells stably transfected with OE Ctrl or OE FZD5 was detected by Western blot and real-time PCR. **E** Viability of Hs-578t cells was analyzed by CCK8. **F** Growth of Hs-578t cells was determined by colony formation, mean ± SD, *n* = 3. *P < 0.05, **P < 0.01, ***P < 0.001, vs. OE Ctrl. **G** Growth curves of xenograft tumors from MDA-MB-231 cells were shown. **H** Phosphorylated histone 3 (p-H3) expression in xenograft tumors was detected by immunohistochemistry. Scalebar: 25 μm, mean ± SD, *n* = 5. **P < 0.01, ***P < 0.001, vs. shCtrl.

Three TNBC cell lines, MDA-MB-231, MDA-MB-468, and Hs-578t with differential FZD5 expression, were selected for the subsequent study. FZD5 expression in MDA-MB-231 cells was stably knockdowned (Fig. 2A). Cell growth in vitro was assessed by CCK8 and colony formation tests. FZD5 knockdown significantly repressed MDA-MB-231 cell growth (Fig. 2B, C). FZD5 knockdown similarly inhibited MDA-MB-468 cell growth (Supplementary Fig. 2A, B). To the contrary, stable FZD5 overexpression remarkably promoted Hs-578t cell growth (Fig. 2D–F). MDA-MB-231 cells with or without FZD5 knockdown were inoculated into nude mice to evaluate the effect of FZD5 on cell growth in vivo. Consistent with the finding in vitro, tumors with FZD5 knockdown grew more slowly compared to tumors without FZD5 knockdown (Fig. 2G). Moreover, tumors with FZD5 knockdown exhibited a lower fraction of phosphorylated Histone 3 (p-H3)-positive and Ki67-positive cells, indicating that FZD5 knockdown blocked cell proliferation in vivo (Fig. 2H, Supplementary Fig. 2C).

### FZD5 promotes G1/S transition and DNA replication

**Fig. 3:**
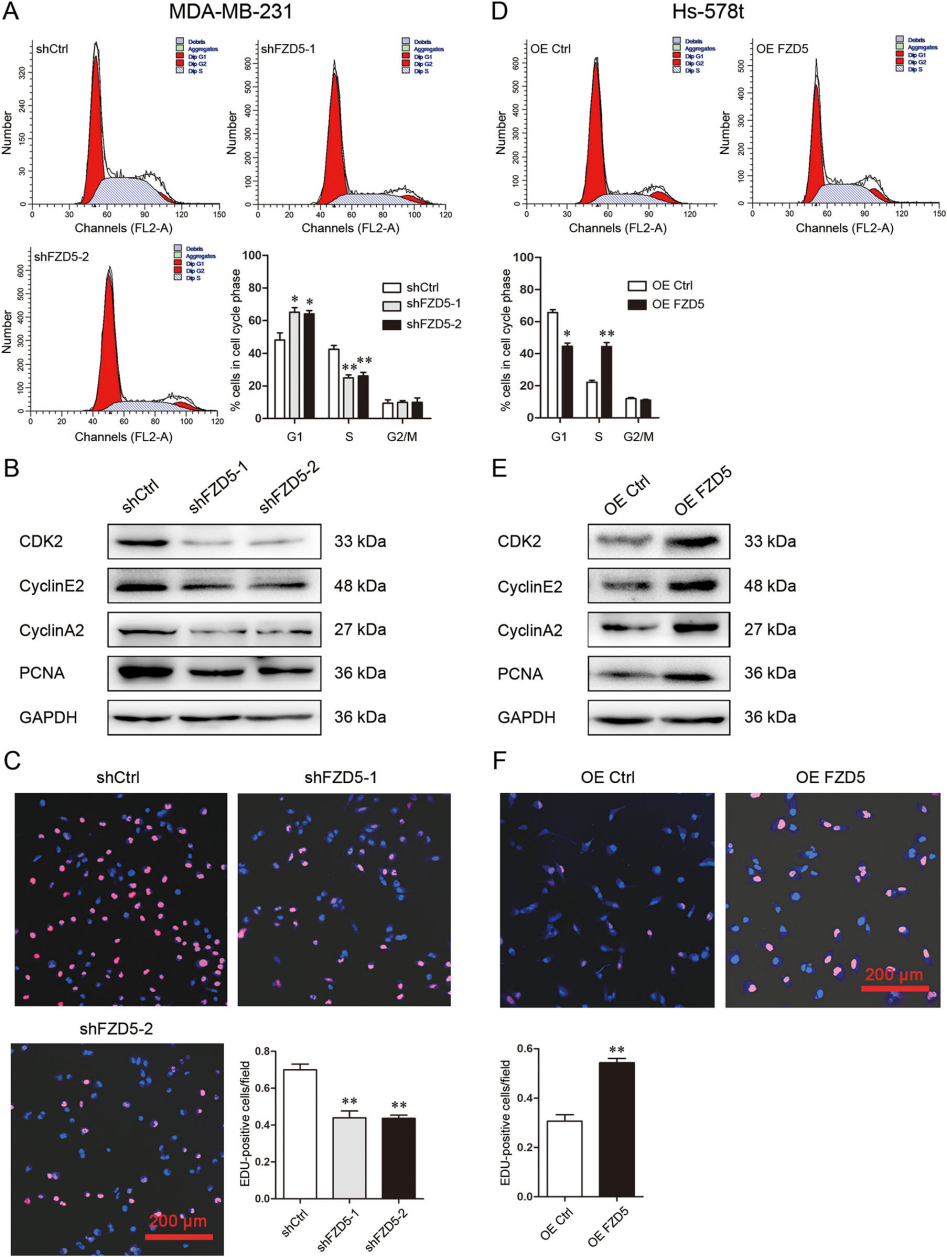
FZD5 promotes G1/S transition and DNA replication. **A** Cell cycle of MDA-MB-231 cells was analyzed by flow cytometry. **B** CDK2, Cyclin E2, Cyclin A2, and PCNA expression in MDA-MB-231 cells was detected by Western blot. **C** DNA replication of MDA-MB-231 cells was analyzed by EDU staining. Scalebar: 200 μm, mean ± SD, *n* = 3. *P < 0.05, **P < 0.01, vs. shCtrl. **D** Cell cycle of Hs-578t cells was analyzed by flow cytometry. **E** CDK2, Cyclin E2, Cyclin A2, and PCNA expression in Hs-578t cells was detected by Western blot. **F** DNA replication of Hs-578t cells was analyzed by EDU staining. Scale bar: 200 μm, mean ± SD, *n* = 3. *P < 0.05, **P < 0.01, vs. OE Ctrl.

Flow cytometry was used to investigate the effect of FZD5 on cell cycle. FZD5 knockdown in MDA-MB-231 and MDA-MB-468 cells increased G1 fraction but lowered S fraction, indicating that FZD5 knockdown arrested G1/S transition (Fig. 3A, Supplementary Fig. 3A). Consistent with this finding, FZD5 knockdown reduced the expression of factors related to G1/S transition such as CDK2, Cyclin E2, and Cyclin A2 (Fig. 3B, Supplementary Fig. 3B). FZD5 knockdown decreased PCNA expression, indicating that DNA replication was suppressed, which was further confirmed by decreased fraction of EDU-positive cells (Fig. 3B, C, Supplementary Fig. 3B, C). Just as expected, FZD5 overexpression in Hs-578t cells diminished G1 fraction but elevated S fraction (Fig. 3D). FZD5 overexpression increased the expression of CDK2, Cyclin E2, Cyclin A2, and PCNA, and the fraction of EDU-positive cells (Fig. 3E, F).

### FZD5 enhances DNA damage repair and chemoresistance

**Fig. 4:**
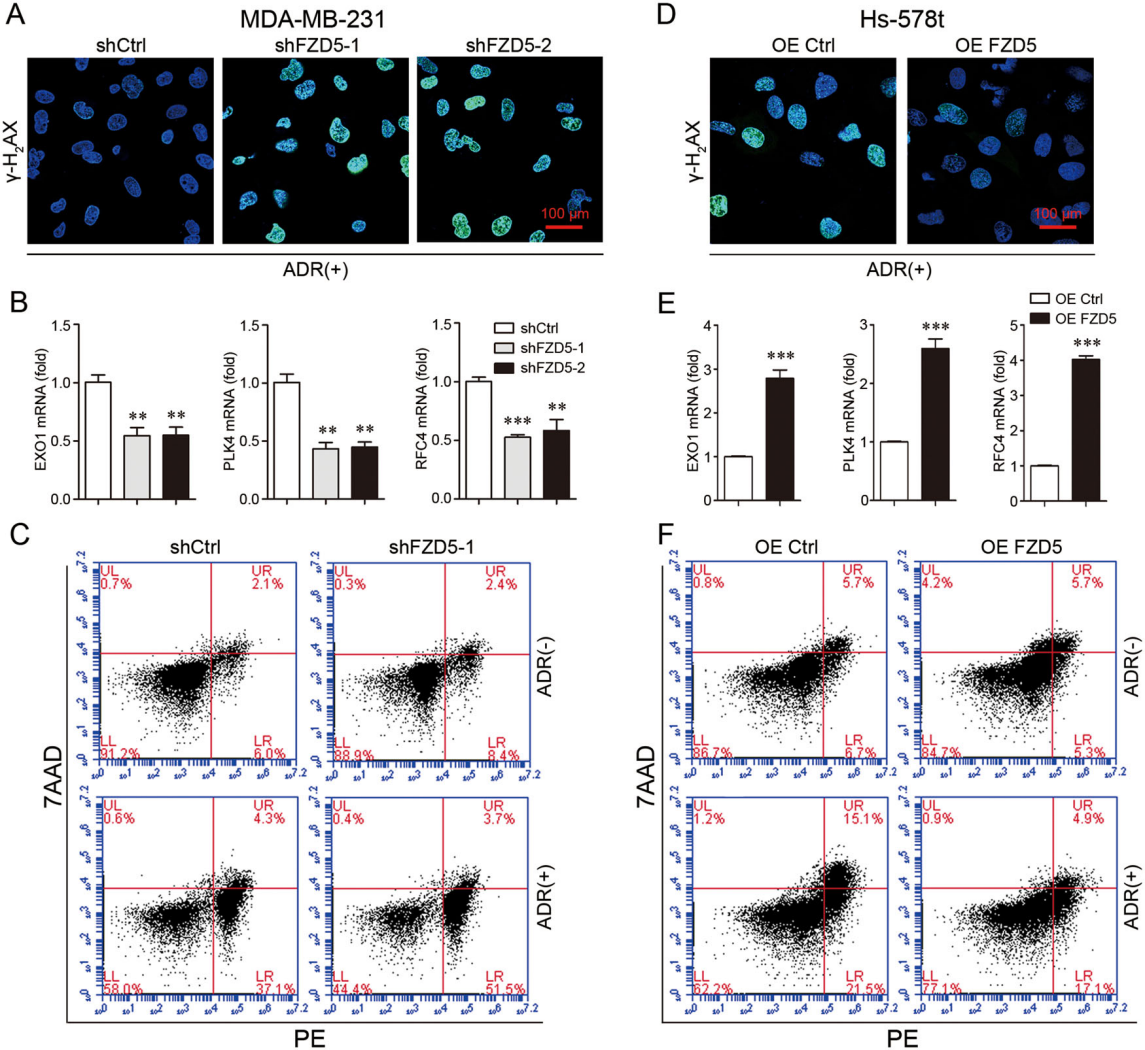
FZD5 enhances DNA damage repair and chemoresistance. **A** γ-H2AX expression in MDA-MB-231 cells was detected by immunofluorescence 48 h after treatment with ADR (300 nM). Scale bar: 100 μm. **B** EXO1, PLK4, and RFC4 expression in MDA-MB-231 cells was detected by real-time PCR, mean±SD, n = 3. **P < 0.01, ***P < 0.001, *vs* shCtrl. **C** Death of MDA-MB-231 cells was detected by flow cytometry 48 h after treatment with ADR (300 nM). **D** γ-H2AX expression in Hs-578t cells was detected by immunofluorescence 48 h after treatment with ADR (300 nM). Scale bar: 100 μm. **E** EXO1, PLK4, and RFC4 expression in Hs-578t cells was detected by real-time PCR, mean ± SD, *n* = 3. ***P < 0.001, vs. OE Ctrl. **F** Death of Hs-578t cells was detected by flow cytometry 48 h after treatment with ADR (300 nM).

As GSEA suggested that FZD5 was related to DNA damage and chemoresistance (Fig. 1D, E, Supplementary Table 1, Supplementary Fig. 1), the role of FZD5 in DNA damage repair and chemoresistance was investigated. Adriamycin (ADR) was used to induce DNA damage, which is characterized by γ-H2AX recruitment. Forty-eight hours after ADR treatment, MDA-MB-231 and MDA-MB-468 cells with FZD5 knockdown still displayed high intensity of γ-H2AX staining, indicating that FZD5 knockdown impaired DNA damage repair (Fig. 4A, Supplementary Fig. 4A). Mechanistically, FZD5 knockdown reduced the expression of several factors related to DNA repair, such as EXO1, PLK4, and RFC4 (Fig. 4B, Supplementary Fig. 4B). An increase in DNA damage repair contributes to chemoresistance, therefore the effect of FZD5 on ADR-induced cell death was determined. FZD5 knockdown elevated the sensitivity of MDA-MB-231 cells to ADR (Fig. 4C). Moreover, FZD5 knockdown increased the sensitivity of MDA-MB-231 cells to Paclitaxel (Supplementary Fig. 5A). FZD5 overexpression, accordingly, promoted DNA damage repair, increased EXO1, PLK4, and RFC4 expression, and induced ADR and Paclitaxel resistance in Hs-578t cells (Fig. 4D–F, Supplementary Fig. 5B).

### FZD5 maintains stem cell-like properties

**Fig. 5:**
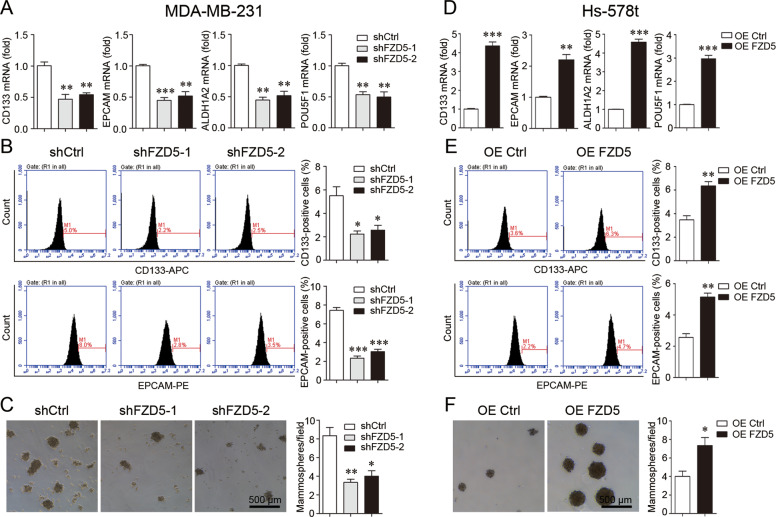
FZD5 maintains stem cell-like properties. **A** CD133, EPCAM, ALDH1A2, and POU5F1 expression in MDA-MB-231 cells was detected by real-time PCR. **B** Fractions of CD133-positive and EPCAM-positive MDA-MB-231 cells were detected by flow cytometry. **C** Mammosphere formation of MDA-MB-231 cells was shown. Scale bar: 500 μm, mean ± SD, *n* = 3. *P < 0.05, **P < 0.01, ***P < 0.001, vs. shCtrl. **D** CD133, EPCAM, ALDH1A2, and POU5F1 expression in Hs-578t cells was detected by real-time PCR. **E** Fractions of CD133-positive and **E**PCAM-positive Hs-578t cells were detected by flow cytometry. **F** Mammosphere formation of Hs-578t cells was shown. Scale bar: 500 μm, mean ± SD, *n* = 3. *P < 0.05, **P < 0.01, ***P < 0.001, vs. OE Ctrl.

DNA damage repair and chemoresistance are associated with cancer cell stem-like traits, therefore the role of FZD5 was further explored. FZD5 knockdown suppressed the expression of a series of stem-related factors including CD133, EPCAM, ALDH1A2, and POU5F1 (OCT4) (Fig. 5A). Consistent with this finding, Flow cytometry showed that FZD5 knockdown reduced the fractions of CD133-positive and EPCAM-positive cells (Fig. 5B, Supplementary Fig. 6). FZD5 knockdown also reduced the fractions of ALDH1-positive cells (Supplementary Fig. 7A, B). FZD5 knockdown weakened the mammosphere formation capacity of breast cancer cells (Fig. 5C). FZD5 overexpression exhibited opposite alterations in Hs-578t cells. FZD5 overexpression upregulated the expression of CD133, EPCAM, ALDH1A2, and POU5F1, increased the fractions of CD133-positive, EPCAM-positive, and ALDH1-positive cells, and enhanced the mammosphere formation capacity (Fig. 5D–F, Supplementary Fig. 7C).

### FOXM1 acts as a downstream effecter of FZD5

**Fig. 6:**
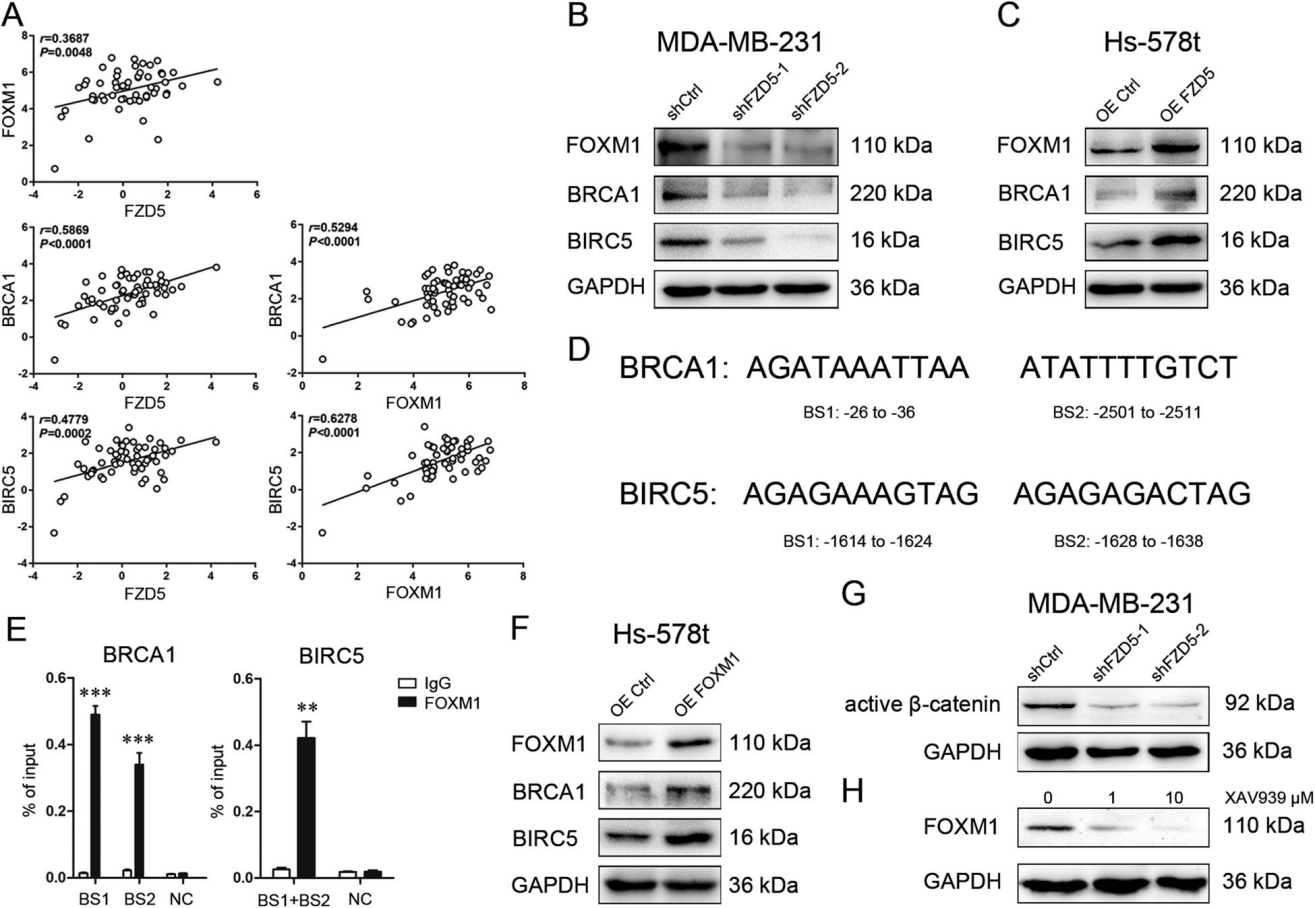
FOXM1 acts as a downstream effecter of FZD5. **A** CCLE database was interrogated for FZD5, FOXM1, BRCA1, and BIRC5 expression. Correlation between two genes in a total of 57 breast cancer cell lines was analyzed by Pearson statistics. **B** FOXM1, BRCA1, and BIRC5 expression in MDA-MB-231 cells was detected by Western blot. **C** FOXM1, BRCA1, and BIRC5 expression in Hs-578t cells was detected by Western blot. **D** Binding sites for FOXM1 on BRCA1 and BIRC5 promoters were shown. **E** Binding of FOXM1 to BRCA1 and BIRC5 promoters in MDA-MB-231 cells was analyzed by ChIP and real-time PCR, mean ± SD, *n* = 3. **P < 0.01, ***P < 0.001, vs. IgG. NC negative control. **F** FOXM1, BRCA1, and BIRC5 expression in Hs-578t cells was detected by Western blot. **G** Active β-catenin expression in MDA-MB-231 cells was detected by Western blot. **H** FOXM1 expression in MDA-MB-231 cells was detected by Western blot.

Interrogation of CCLE database revealed a positive correlation of FZD5 with FOXM1, BRCA1, and BIRC5, several key factors related to cell cycle, DNA replication, DNA damage repair, and survival, in a total of 57 breast cancer cell lines (Fig. 6A, Supplementary Table 2). These correlations also existed in 28 TNBC cell lines (Supplementary Table 3, Supplementary Fig. 8). Loss and gain of function studies confirmed that FZD5 modulated these factors in breast cancer cells (Fig. 6B, C). Whether FOXM1, a transcription factor, promoted BRCA1 and BIRC5 gene transcription was subsequently determined. The binding sites for FOXM1 were identified in BRCA1 and BIRC5 promoters (Fig. 6D). ChIP in combination with real-time PCR detection verified that FOXM1 could bind to these two gene promoters (Fig. 6E). Furthermore, FOXM1 overexpression in Hs-578t cells induced BRCA1 and BIRC5 expressions (Fig. 6F). FZD5 knockdown reduced active β-catenin level, and treatment with β-catenin inhibitor XAV939 suppressed FOXM1 expression, indicating that FZD5 modulated FOXM1 in a Wnt/β-catenin-dependent manner (Fig. 6G, H).

### FOXM1 overexpression induces FZD5-associated phenotype

**Fig. 7:**
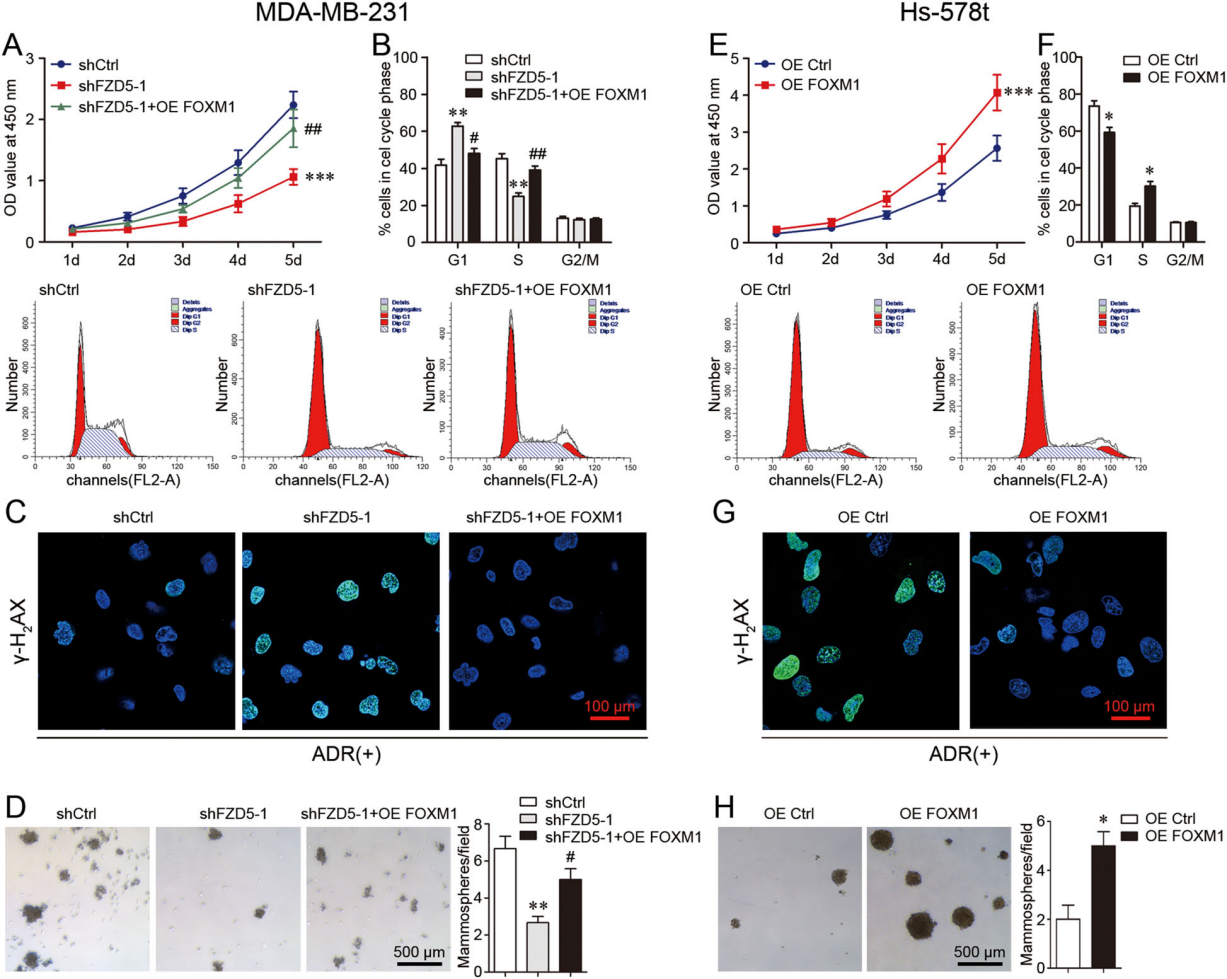
FOXM1 overexpression induces FZD5-associated phenotype. **A** Viability of MDA-MB-231 cells was detected by CCK8. **B** Cell cycle of MDA-MB-231 cells was analyzed by flow cytometry. **C** γ-H2AX expression in MDA-MB-231 cells was detected by immunofluorescence 48 h after treatment with ADR (300 nM). Scale bar: 100 μm. **D** Mammosphere formation of MDA-MB-231 cells was shown. Scale bar: 500 μm, mean ± SD, *n* = 3. **P < 0.01, ***P < 0.001, vs. shCtrl; ^#^*P* < 0.05, ^##^*P* < 0.01, vs. shFZD5-1. **E** Viability of Hs-578t cells was detected by CCK8. **F** Cell cycle of Hs-578t cells was analyzed by flow cytometry. **G** γ-H2AX expression in Hs-578t cells was detected by immunofluorescence 48 h after treatment with ADR (300 nM). Scale bar: 100 μm. **H** Mammosphere formation of Hs-578t cells was shown. Scale bar: 500 μm, mean ± SD, *n* = 3. *P < 0.05, ***P < 0.001, vs. OE Ctrl.

To verify FOXM1 as a functional downstream molecule of FZD5 signaling, FOXM1 was overexpressed in MDA-MB-231 cells with FZD5 knockdown. FOXM1 overexpression increased cell viability, promoted G1/S transition, enhanced DNA damage repair, and induced mammosphere formation, indicating that FOXM1 overexpression restored FZD5-associated phenotype (Fig. 7A–D). Moreover, FOXM1 overexpression induced FZD5-associated phenotype in FZD5-low Hs-578t cells (Fig. 7E–H). Together, these results demonstrated the role of FZD5-FOXM1 signaling in cell cycle, DNA replication, DNA damage repair, and stem-like properties.

### Wnt7B is involved in cell proliferation, DNA damage repair, and stemness

**Fig. 8:**
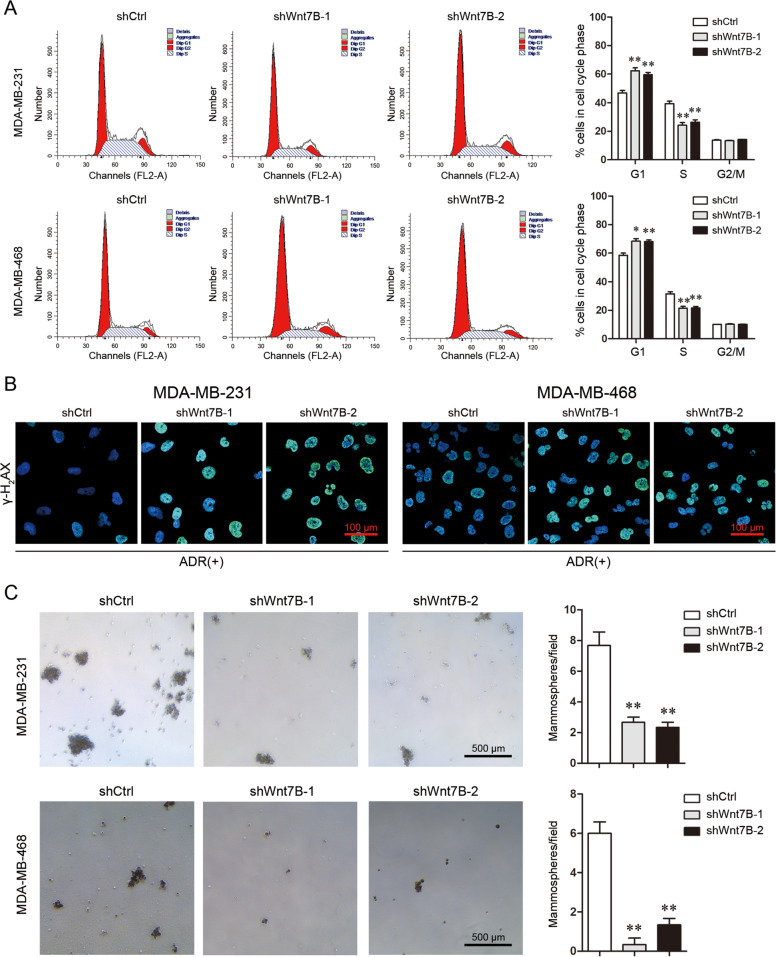
Wnt7B is involved in cell proliferation, DNA damage repair, and stemness. **A** Cell cycle of MDA-MB-231 and MDA-MB-468 cells was analyzed by flow cytometry. **B** γ-H2AX expression in MDA-MB-231 and MDA-MB-468 cells was detected by immunofluorescence 48 h after treatment with ADR (300 nM). Scale bar: 100 μm. **C** Mammosphere formation of MDA-MB-231 and MDA-MB-468 cells was shown. Scale bar: 500 μm, mean ± SD, *n* = 3. *P < 0.05, **P < 0.01, vs. shCtrl.

Wnt7B is a specific ligand for FZD5^25,26^. Wnt7B knockdown in MDA-MB-231 and MDA-MB-468 cells decreased cell viability and arrested G1/S transition, indicating that Wnt7B downregulation suppressed cell proliferation (Supplementary Fig. 9A, B, Fig. 8A). MDA-MB-231 and MDA-MB-468 cells with Wnt7B knockdown still displayed high intensity of γ-H2AX staining after ADR treatment, demonstrating that Wnt7B downregulation impaired DNA damage repair (Fig. 8B). Furthermore, Wnt7B knockdown weakened the mammosphere formation capacity of both types of breast cancer cells (Fig. 8C). These data suggest that contribution of FZD5 to breast cancer cell growth and chemoresistance is at least in part dependent on Wnt7B.

## Discussion

FZD belong to the superfamily of G protein-coupled receptor (GPCR). The N-terminus contains a cysteine-rich domain (CRD), via which FZD can bind Wnt ligands. The C-terminus binds Dishevelled (Dvl) and interacts with G proteins. 10 FZD have thus far been identified in human. FZD5 induces proliferation of cancer cells by activating β-catenin pathway^25^. FZD5 also mediates β-catenin-independent pathways to increase tumor cell motility^27^. Our study revealed a novel role of FZD5 in TNBC. FZD5 initiates a signaling to enhance DNA damage repair and induce chemoresistance. FZD5 promotes Cyclin A, Cyclin E, CDK2 and PCNA expression, G1/S transition and DNA replication. Furthermore, FZD5 is associated with epithelial-like stemness characterized by hyper-proliferation and higher expression of ALDH1, EPCAM, and CD133^13,14^. TNBC is characterized by aberrant activation of both canonical and non-canonical Wnt pathways, which induces TNBC stemness and metastasis^28,29^. Several other FZDs such as FZD6 and FZD7 have been shown implicated in TNBC^30,31^.

Our study identified FOXM1 as a downstream effecter of FZD5 signaling. FOXM1 is one of the members of the Forkhead family proteins. In normal cells, FOXM1 regulates cell cycle transition. Overexpression of FOXM1 has been observed in a variety of cancer types, indicating that FOXM1 plays essential roles in carcinogenesis^32,33^. In breast cancer, RNA-Seq analysis has revealed that FOXM1 is associated with not only proliferation, but also cell cycle transition, apoptosis, regulation of transcription, DNA replication, and DNA damage repair^34^. FOXM1 contributes to chemoresistance in breast cancer by enhancing DNA damage repair^35–38^. Moreover, suppression of FOXM1 was shown to reduce breast cancer growth in vitro and in vivo^34,39^.

BRCA1 plays a crucial role in the maintenance of DNA stability^40^. Therefore, BRCA1 mutation results in increased genomic instability and risk of developing breast and ovarian cancers due to deficient DNA repair^41^. About 20% TNBC harbor BRCA1/2 mutation, and patients with these tumors have a significantly lower risk of recurrence^42^. The better prognosis may largely be related to the higher sensitivity to anticancer drugs due to deficient DNA damage repair. To the contrary, increased DNA repair capacity contributes to chemoresistance. High BRCA1 expression is correlated with chemoresistance in hepatocellular carcinoma^43^. Tamoxifen-resistant breast cancer cells are resistant to cisplatin and ADR because of high BRCA1 expression^44^. Our study has identified FZD5-FOXM1 signaling as an upstream modulator of BRCA1, which mediates FZD5-induced chemoresistance in TNBC cells without BRCA1 mutation.

BIRC5/Survivin is one of the members of the inhibitor of apoptosis protein family (IAPs). High BIRC5 expression in tumor cells correlates with cell division, apoptosis inhibition, chemoresistance, and stemness^45^. BIRC5 downregulation inhibits cell growth but promotes apoptosis in breast cancer cells^46^. Moreover, BIRC5 silencing induces DNA double-strand breaks and impairs DNA repair capacity^47^. BIRC5 is transcriptionally modulated by FOXM1, and overexpression of FOXM1 and BIRC5 is related to drug resistance and unfavorable prognosis in breast cancer^38^. BIRC5 silencing also inhibits self-renewal and chemoresistance of EPCAM-positive breast CSC^48^.

In summary, our study has demonstrated a novel function of FZD5 in breast cancer, especially in TNBC. FZD5 contributes to G1/S transition, DNA replication, DNA damage repair, chemoresistance, and stemness. As a downstream effector of FZD5 signaling, FOXM1 transcriptionally upregulates BRCA1 and BIRC5, both of which play crucial roles in these processes. Therefore, FZD5-FOXM1 may be a potential target for the development of strategies to increase chemosensitivity and prevent recurrence in TNBC.

## Materials and methods

### In silico analysis

TCGA (TNBC: 115; non-TNBC: 858) and GSE2603 (TNBC: 25; non-TNBC: 71) databases were interrogated for FZD5 mRNA expression in breast cancer samples. The association of FZD5 with survival was analyzed in Kaplan–Meier plotter website (http://kmplot.com/analysis/)^49^. GSEA was performed using TCGA database. According to the median value of FZD5 mRNA level, the samples were divided into FZD5-high and FZD5-low groups. The gene pathways differentially expressed between FZD5-high and FZD5-low breast cancer samples were analyzed. Cancer Cell Line Encyclopedia (CCLE) database was interrogated for FZD5, FOXM1, BRCA1, and BIRC5 mRNA expression in a series of human breast cancer cell lines (Supplementary Tables 2 and 3). Correlation between two genes was analyzed by Pearson statistics.

### Human specimens

18 TNBC and 24 non-TNBC samples were obtained from Cancer Hospital of China Medical University with the informed consent of the patients. Institutional Research Ethics Committee of China Medical University approved the use of these tissues for research purposes.

### Immunohistochemistry

The tissues were fixed in 4% paraformaldehyde for 72 h, embedded in paraffin and then sliced into 4 μm sections. Xylene and gradient alcohol were used to deparaffinize and hydrate, respectively. 3% H_2_O_2_ was used to eliminate endogenous peroxidase activity. Citrate buffer was used to repair antigen, and then the sections were blocked by BSA. The sections were incubated with primary antibodies (anti-FZD5, Abcam, ab75234, UK, 1:200; anti-phosphorylated Histone 3, ThermoFisher, PA5-17869, USA, 1:200; anti-Ki67, Invitrogen, 14-5699-95, USA, 1:500) overnight at 4 °C. Subsequently, the sections were incubated with goat anti-rabbit IgG and streptavidin peroxidase (SP) complex at 37 °C for 30 min, and stained with DAB reagent. Finally, the sections were re-stained with hematoxylin, dehydrated with gradient alcohol and xylene, and observed under a microscope (LEICA DM2500 LED). Phosphorylated Histone 3-positive or Ki67-positive cells in five randomly selected fields were counted.

### Cell culture and transfection

MDA-MB-231, MDA-MB-468, and Hs-578t cells were obtained from the Nanjing KeyGen Biology (Nanjing, China). All human cell lines have been authenticated using STR profiling. MDA-MB-231 and Hs-578t cells were cultured in DMEM (Hyclone). MDA-MB-468 was cultured in Leibovitz’s L-15 (Hyclone). The culture media were supplemented with 10% fetal bovine serum (FBS). All cells were cultured at 37 °C with 5% CO_2_ in a humidified incubator. MDA-MB-231 and MDA-MB-468 cells were transfected with shFZD5 or shWnt7B lentiviruses (GV112/hU6-MCS-CMV-Puromycin, Genechem, China) to stably knockdown FZD5 or Wnt7B expression. Hs-578t cells were transfected with FZD5 overexpression lentiviruses (GV492/Ubi-MCS-3FLAG-CBh-gcGFP-IRES-Puromycin, Genechem, China) to stably overexpress FZD5. After infection for 48 h, cells were selected by 2 μg/mL puromycin (Sigma). In some experiments, MDA-MB-231 and Hs-578t cells were transfected with FOXM1 overexpression plasmids (GV219/CMV-MCS-SV40-Neomycin, Genechem, China) using Lipofectamine 3000 in Opti-MEM medium according to the product manual. The target sequence for shFZD5-1 is 5′-CGGCATCTTCACGCTGCTCTA-3′, for shFZD5-2 is 5′-GGCCACCTTCCTCATCGACAT-3′, for shWnt7B-1 is 5′-gcGCCTCATGAACCTGCATAA-3′, for shWnt7B-2 is 5′-cgTGCGTTACGGCATCGACTT-3′, and for control is 5′-TTCTCCGAACGTGTCACGT-3′.

### Western blot

Cells were lysed using RIPA lysis buffer with 1% PMSF on ice for 1 h. Protein fragments were centrifuged with 12,000 × *g* at 4 °C for 40 min. A BCA protein assay kit was used to determine the protein concentration. 30 μg protein was separated on 10% SDS–PAGE gel and transferred into a polyvinylidene difluoride (PVDF) membrane in a wet electron transfer device. The membrane was blocked in 5% skimmed milk in Tris-buffered saline (TBS) containing 0.05% Tween 20 for 2 h at room temperature. Subsequently, the membrane was incubated with various primary antibodies at 4 °C overnight. After being washed in TBST three times, the membrane was incubated in horseradish peroxidase (HRP)-conjugated goat anti-rabbit for 1.5 h at room temperature. The primary antibodies are as follows: FZD5 (Cell Signaling Technology, #5266, USA, 1:1000), BRCA1 (Cell Signaling Technology, #9010, USA, 1:1000), CDK2 (Cell Signaling Technology, #2546, USA, 1:1000), Cyclin E2 (Cell Signaling Technology, #4132, USA, 1:1000), active β-catenin (Cell Signaling Technology, #8814, USA, 1:1000), Cyclin A2 (SANTA, sc-53234, USA, 1:1000), PCNA (SANTA, sc-71858, USA, 1:1000), BIRC5 (ThermoFisher, PA5-16859, USA, 1:1000), FOXM1 (ThermoFisher, PA5-71455, USA, 1:1000), Wnt7B (Abcam, ab94915, UK, 1:1000). An enhanced chemiluminescene (ECL) kit was used to visualize target protein.

### Quantitative real-time PCR

Total RNA was extracted from cells with TRIZOL Reagent (Takara, 9108/9109, China) according to the standard instructions. Reverse transcription was conducted with the cDNA synthesis Kit (Takara, RR047A, China) with 1 μg RNA. The target cDNA was amplified by TB Green™ *Premix Ex Taq* II and an ABI PRISM 7300 Sequence Detection system (Applied Biosystems, USA). The relative gene expression was analyzed using the 2^−ΔΔCt^ method. GAPDH was used as control. The primers used are listed in Table 1.

**Table 1:** Primers for real-time PCR.

Genes or binding sites	Primers (5′–3′)
FZD5-forward	TCCTCTGCATGGATTACAACC
FZD5-reverse	GACACTTGCACACGAACG
EXO1-forward	GCTCGGCTAGGAATGTGCAGAC
EXO1-reverse	CCCACGCAGTGATGACAGGTAG
PLK4-forward	CCTTCTCAGAAAATGAAGCTCG
PLK4-reverse	TCATGTGGCATTTTCAGTTGAG
RFC4-forward	AAACCACCCGATTCTGTCTTAT
RFC4-reverse	CTTGGCAATGTCTAGTAATCGC
CD133-forward	GTGGCGTGTGCGGCTATGAC
CD133-reverse	CCAACTCCAACCATGAGGAAGACG
EPCAM-forward	GTCTGTGAAAACTACAAGCTGG
EPCAM-reverse	CAGTATTTTGTGCACCAACTGA
ALDH1A2-forward	TGCTGATGCTGACTTGGACTATGC
ALDH1A2-reverse	CCGCTCCACGCTTCTTCTCAC
POU5F1-forward	GATGTGGTCCGAGTGTGGTTCTG
POU5F1-reverse	CGAGGAGTACAGTGCAGTGAAGTG
GAPDH-forward	CAGGAGGCATTGCTGATGAT
GAPDH-reverse	GAAGGCTGGGGCTCATTT
BRCA1 BS1-forward	CTTTTACGTCATCCGGGGGC
BRCA1 BS1-reverse	CGCGCAGTCGCAGTTTTAAT
BRCA1 BS2-forward	GCAGTGGTGCAATCTGGG
BRCA1 BS2-reverse	GGTGGATCACGAGGTCAAG
BIRC5 BS1+BS2-forward	TGGTAATGCCTTCAACTT
BIRC5 BS1+BS2-reverse	TCTCCCCAACCTACTTTC

### Cell viability assay

Cells were trypsinized and seeded into 96-well plates (MDA-MB-231: 5 × 10^3^ cells/well; MDA-MB-468: 5 × 10^3^ cells/well; Hs-578t: 2 × 10^3^ cells/well). 10 μL Cell Counting Kit-8 (Dojindo Molecular Technologies, Japan) was added into each well and incubated for 1 h at 37 °C. The absorbance was measured at 450 nm by a microplate reader (Bio-Rad Laboratories, USA) at different time points.

### Colony formation assay

Cells were trypsinized and cultured in 3.5 cm plates (MDA-MB-231: 2 × 10^3^ cells/well; Hs-578t: 2 × 10^3^ cells/well) in medium with 10% FBS containing 5% CO_2_ for 2 weeks. The cells were then fixed with 4% paraformaldehyde for 15 min and stained with 1% crystal violet for 30 min at 37 °C. Colonies were counted and photographed.

### In vivo animal study

Female BALB/c nude mice (5–6 weeks of age, 18–20 g) were purchased from Weitong Lihua (Beijing, China), and all animals were dealt with according to the Animal Ethics Committee of China Medical University. Before tumor cell inoculation mice were randomized into different groups (five in each group). 5 × 10^6^ MDA-MB-231 cells were resuspended in 100 μL PBS with 50% Matrigel, and injected into the mammary fat pad of the mice. Tumor length and width were measured with a vernier caliper every 3 days. Tumor volume was calculated by the formula: *V* = 1/2 × length × width^2^. The investigator was blinded to the group allocation of the animals during the experiment.

### Cell cycle assay

1 × 10^6^ cells were harvested and washed with PBS, then fixed with 70% ethanol at 4 °C overnight. All samples were treated with 5 μL RNase A and 450 μL propidium iodide (PI) (KeyGEN BioTECH) for 1 h at room temperature in the dark and analyzed by a FACS calibur flow cytometer (BD).

### EDU staining

Cells were seeded in 24-well plates (MDA-MB-231: 5 × 10^5^/well; MDA-MB-468: 5 × 10^5^/well; Hs-578t: 4 × 10^5^/well) for 24 h, and then incubated with 50 μM 5-Ethynyl-20-deoxyuridine (EDU, Ribobio) for 2 h according to the manufacturer’s instruction. Subsequently, cells were washed with PBS, fixed with 4% paraformaldehyde for 30 min and permeabilized with 0.5% Triton X-100 for 10 min. Cells were then treated with 200 μL 1 × Apollo reaction cocktail for 30 min at room temperature in the dark. At last, the DNA contents were stained by 200 μL 1 × Hoechst 33342 for 30 min at room temperature in the dark. A Laser scanning confocal focus microscope was used to visualize the staining. Positive cells in random five fields were counted.

### Immunofluorescence

Cells were seeded in 24-well plates (MDA-MB-231: 5 × 10^5^/well; MDA-MB-468: 5 × 10^5^/well; Hs-578t: 4 × 10^5^/well). After incubation for 24 h, ADR (Solarbio, China) was added into cell cultures at a final concentration of 300 nM for 48 h. The cells were washed with PBS, fixed with 4% paraformaldehyde at room temperature for 30 min, permeabilized with 0.5% Triton X-100 at room temperature for 10 min, and blocked with 5% donkey serum and 0.3% Triton X-100 in PBS at room temperature for 1 h. The cells were then incubated with antibody for γ-H_2_AX (Cell Signaling Technology, #9718, USA, 1:400) at 4 °C overnight. Subsequently, the cells were incubated by Alexa Fluor 488-conjugated anti-mouse IgG for 2 h at room temperature in the dark. The nuclei were stained by DAPI at room temperature for 5 min. Immunofluorescence staining was observed under a laser scanning confocal focus microscope.

### Apoptosis assay

5×10^5^ cells were harvested and washed with PBS. According to the standard protocol, an Annexin V-PE/7-AAD Apoptosis Kit (KeyGEN BioTECH, China) was used to analyze cell apoptosis. Cells were incubated in 500 μL binding buffer with 5 μL 7-AAD at room temperature in the dark for 15 min. The number of apoptotic cells was measured by a FACS Calibur Flow Cytometer (BD).

### Cell subpopulation assay

CD133 and EPACM positive cells were detected by flow cytometry (Calibur Flow Cytometer, BD). 1 × 10^6^ cells were harvested and washed with PBS for three times. 1 μg allophycocyanin (APC)-labeled antibody for CD133 (Invitrogen, 17-1338-42, USA) or phycoerythrin (PE)-labeled antibody for EPCAM (Invitrogen, 12-9326-42, USA) was incubated for 20 min on ice in the dark, and cells were resuspended in 100 μL fluorescence-activated cell sorting (FACS) buffer. ALDH1-positive cells were also detected by FACS using ALDEFLUOR^TM^ Kit (STEMCELL Technologies, USA) according to its standard protocol.

### Tumorisphere formation

1 × 10^4^ cells were seeded in a six-well attachment surface polystyrene culture plate (Corning Costar, USA). Cells were cultured in complete MammoCult™ Human Medium (STEMCELL Technologies, USA) at 37 °C and 5% CO_2_ for 12 days. Spheroids in five randomly selected fields were counted.

### Chromatin immunoprecipitation (ChIP)

ChIP was analyzed using an assay kit (Beyotime, China) according to the manufacturer’s protocols. Briefly, Cells in 10 cm plates were fixed with 1% formaldehyde for 10 min at 37 °C. To shear the chromatin, cells were treated with 1 mM PMSF in SDS lysis buffer for 10 min at 4 °C, followed by cell sonication for 15 min at 4 °C. After a portion of the cross-linked chromatin was removed as input for the subsequent test, the remaining cell lysis was incubated with 1 μg anti-FOXM1 antibody at 4 °C overnight. Then protein A + G agarose was added to precipitate the target protein recognized by anti-FOXM1 antibody for 1 h at 4 °C. Anti-IgG antibody was used as a negative control (NC). The beads were then washed off and DNA was collected for subsequent real-time PCR. The enrichment was indicated as % of input. The NC sequence for BRCA1 is AGACAGTAACT, and for BIRC5 is GAGAAGTGAG. The primers for BRCA1 NC are CTAACATGGCGGACAAAGACA (forward) and GAGGGACAAGTGGTAAGAGCC (reverse), for BIRC5 NC are GGGGCTGGAGGGCTAATA (forward) and TGCTTTGGAACAGGGTGT (reverse).

### Statistical analysis

All cell experiments were performed in triplicate. The data are expressed as mean ± SD. GraphPad prism 5 was used to analyze the data. Differences were analyzed by two-sided Student’s *t* test or one-way ANOVA when the variance is similar between the groups. *P* value < 0.05 was considered statistically significant. No statistical method was used to predetermine the sample size for xenograft mice experiment, which was based on previous experimental observations. The sample size of each experiment is shown in the legend. No data was excluded from the analysis.

## Supplementary information

Supplementary-Figure 1

Supplementary-Figure 2

Supplementary-Figure 3

Supplementary-Figure 4

Supplementary-Figure 5

Supplementary-Figure 6

Supplementary-Figure 7

Supplementary-Figure 8

Supplementary-Figure 9

Supplementary Figure Legends

Supplementary-Table 1

Supplementary-Table 2

Supplementary-Table 3
