# Supplementary figures and images for: FZD5 contributes to TNBC proliferation, DNA damage repair and stemness
Source: Cell Death Dis. 2020 Dec 12;11(12):1060. doi: 10.1038/s41419-020-03282-3 (PMC7733599; doi:10.1038/s41419-020-03282-3)

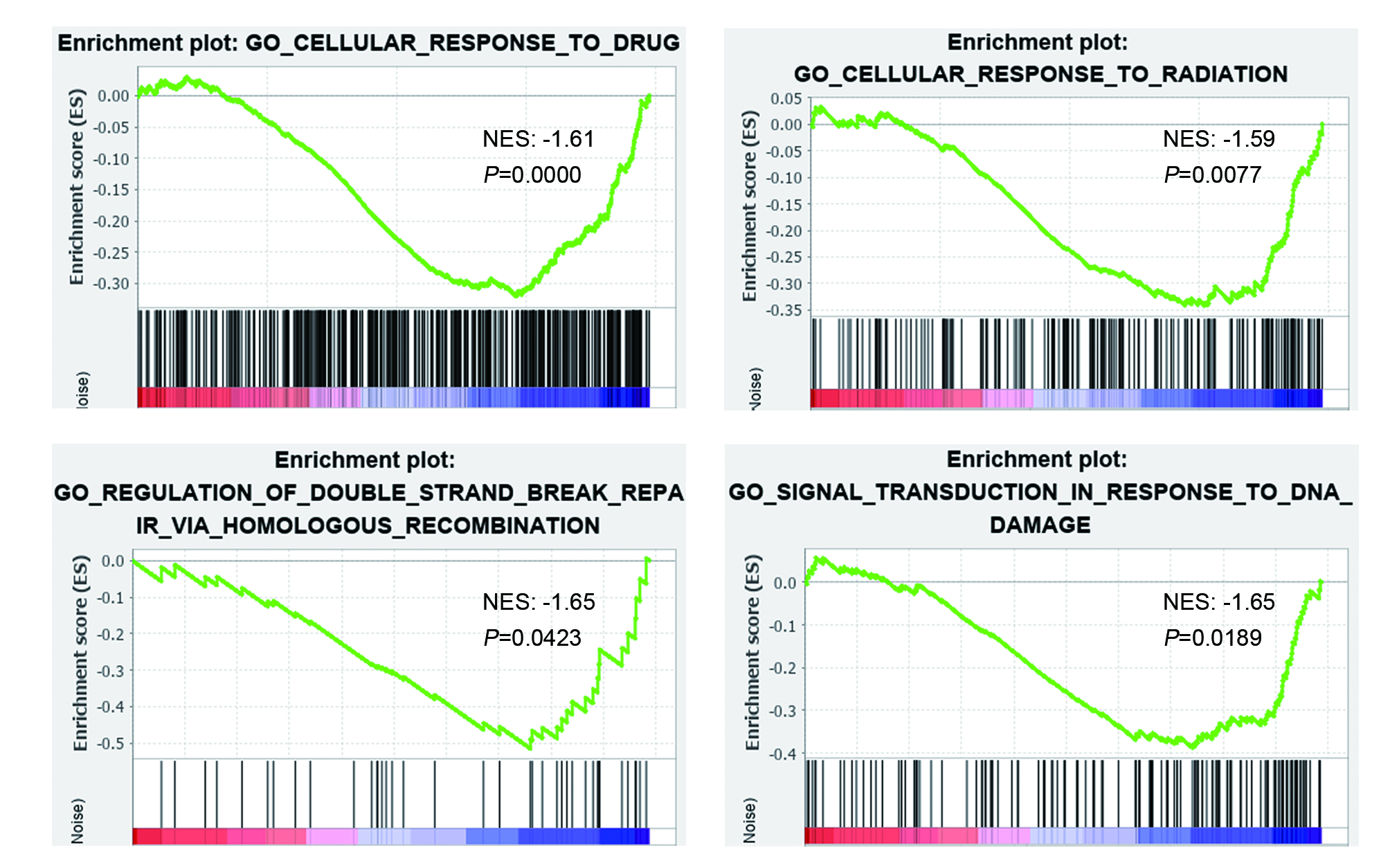

Supplement: Supplementary file 1 — Supplementary-Figure 1 [file 41419_2020_3282_MOESM1_ESM.tif]

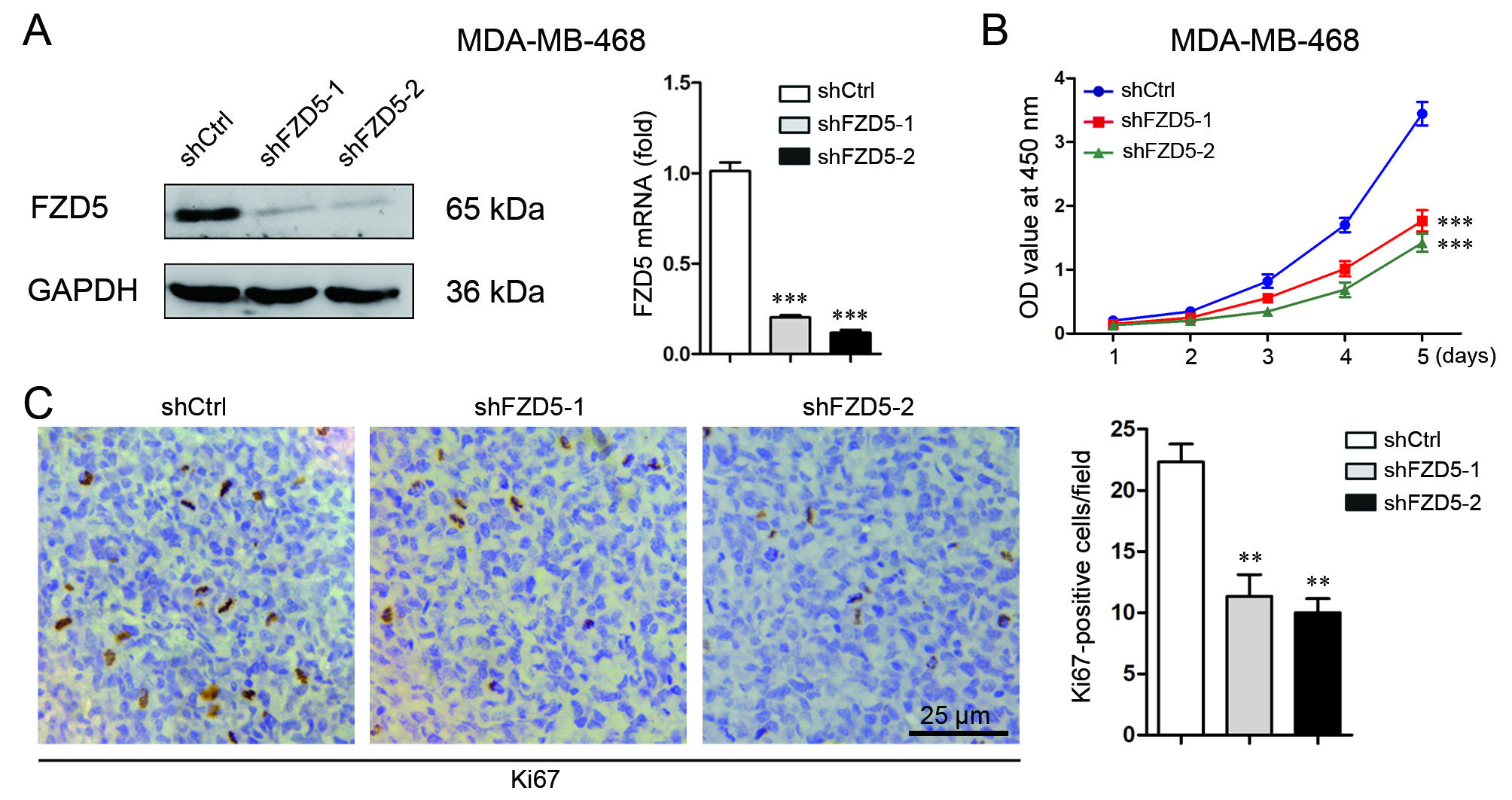

Supplement: Supplementary file 2 — Supplementary-Figure 2 [file 41419_2020_3282_MOESM2_ESM.tif]

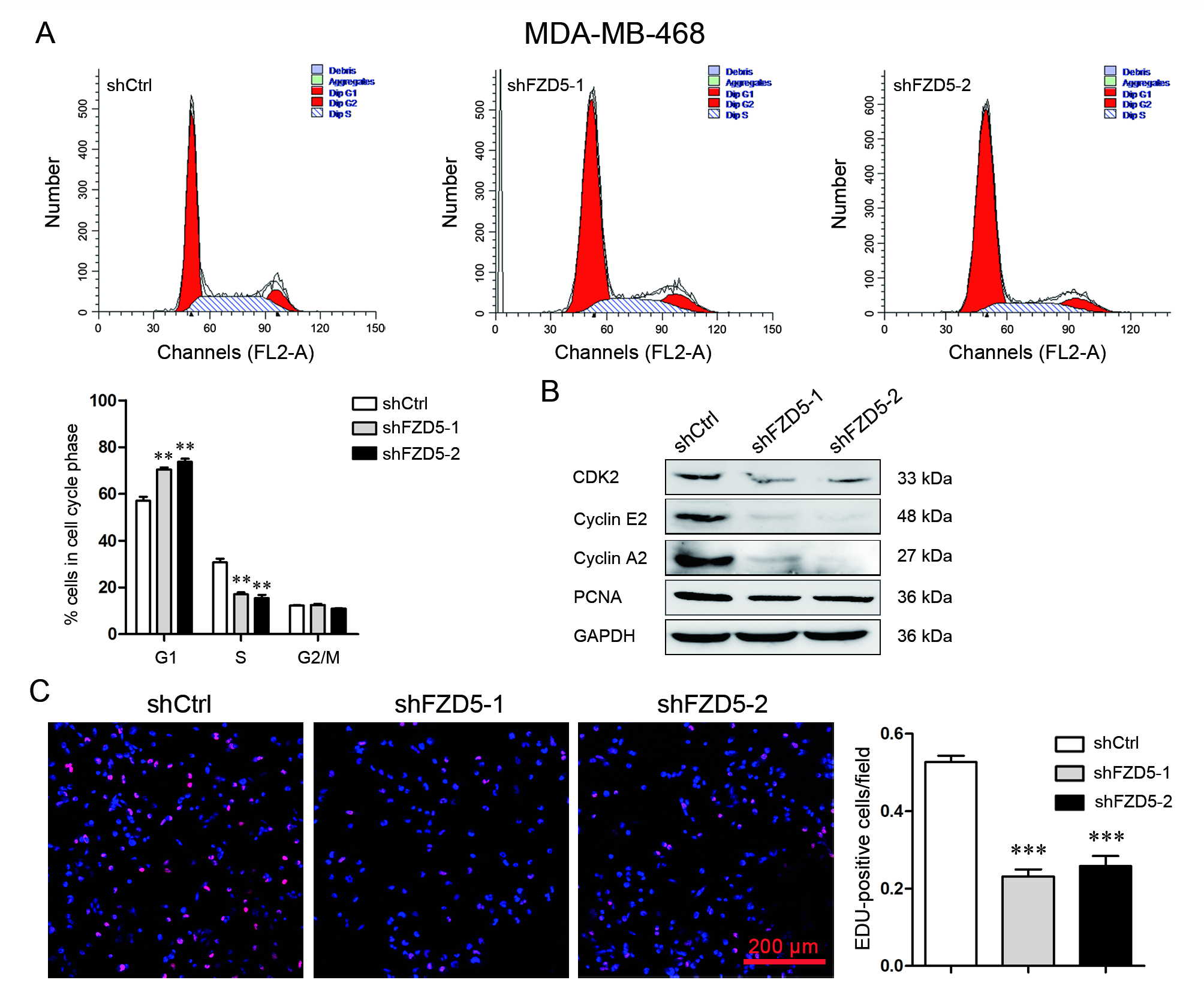

Supplement: Supplementary file 3 — Supplementary-Figure 3 [file 41419_2020_3282_MOESM3_ESM.tif]

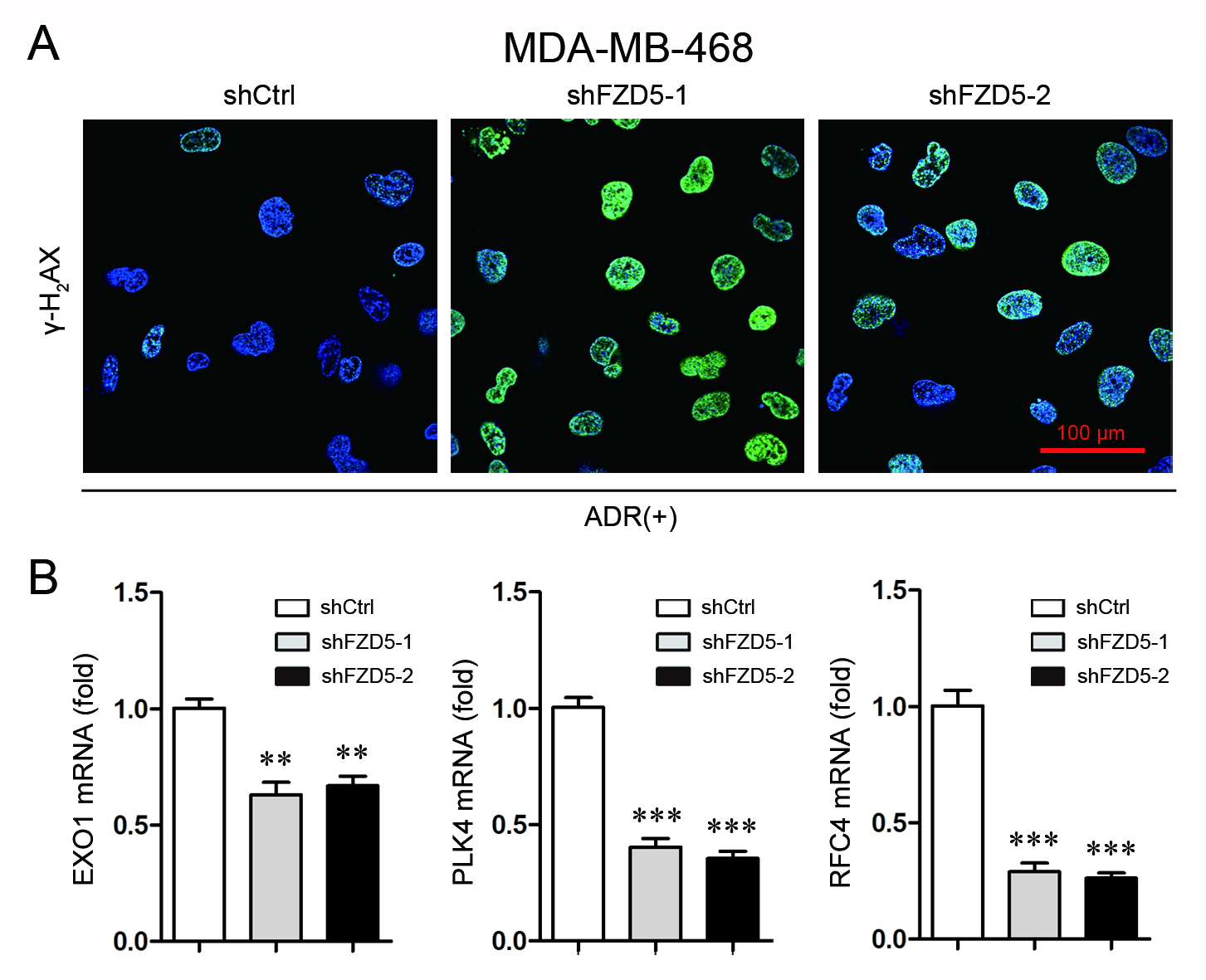

Supplement: Supplementary file 4 — Supplementary-Figure 4 [file 41419_2020_3282_MOESM4_ESM.tif]

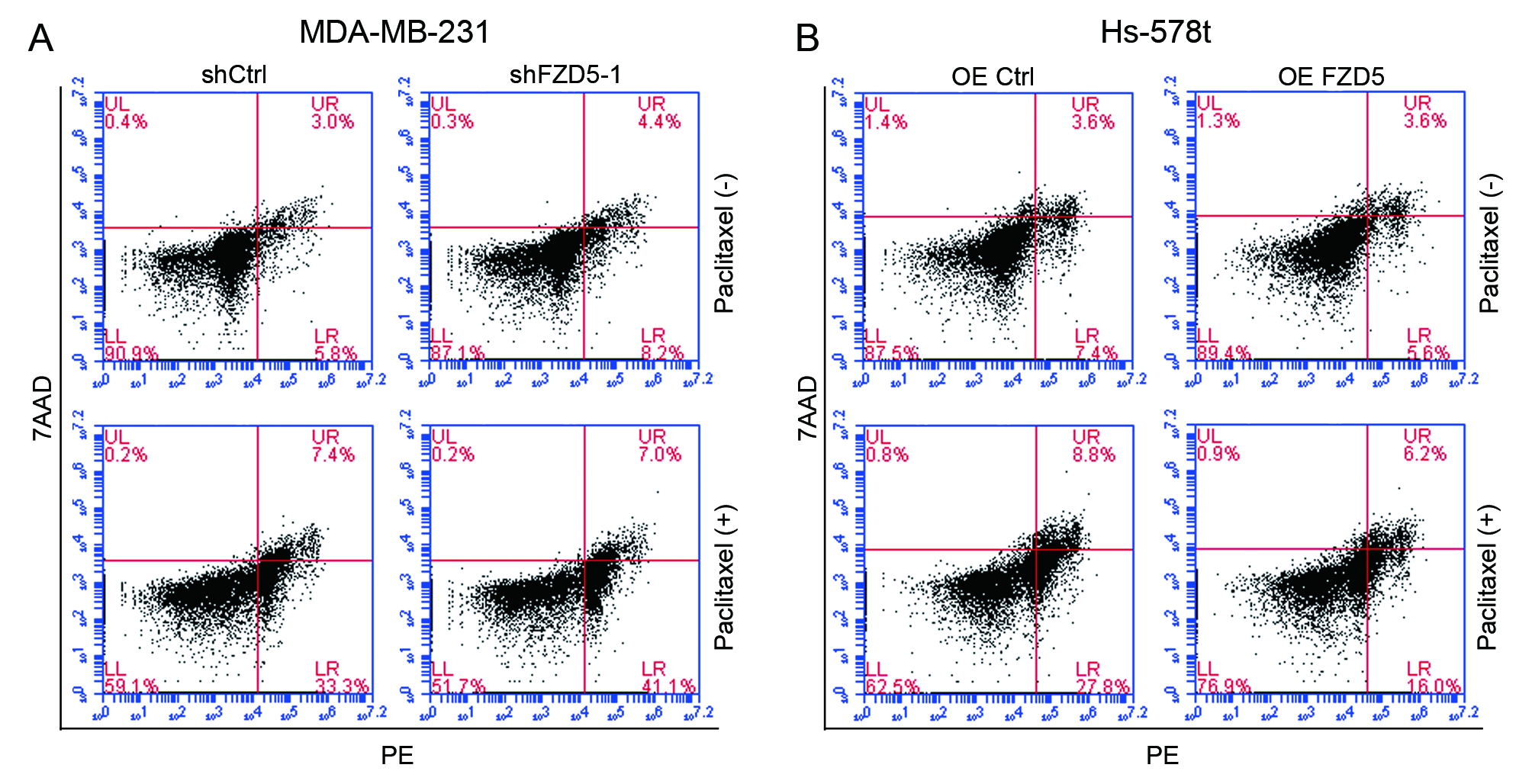

Supplement: Supplementary file 5 — Supplementary-Figure 5 [file 41419_2020_3282_MOESM5_ESM.tif]

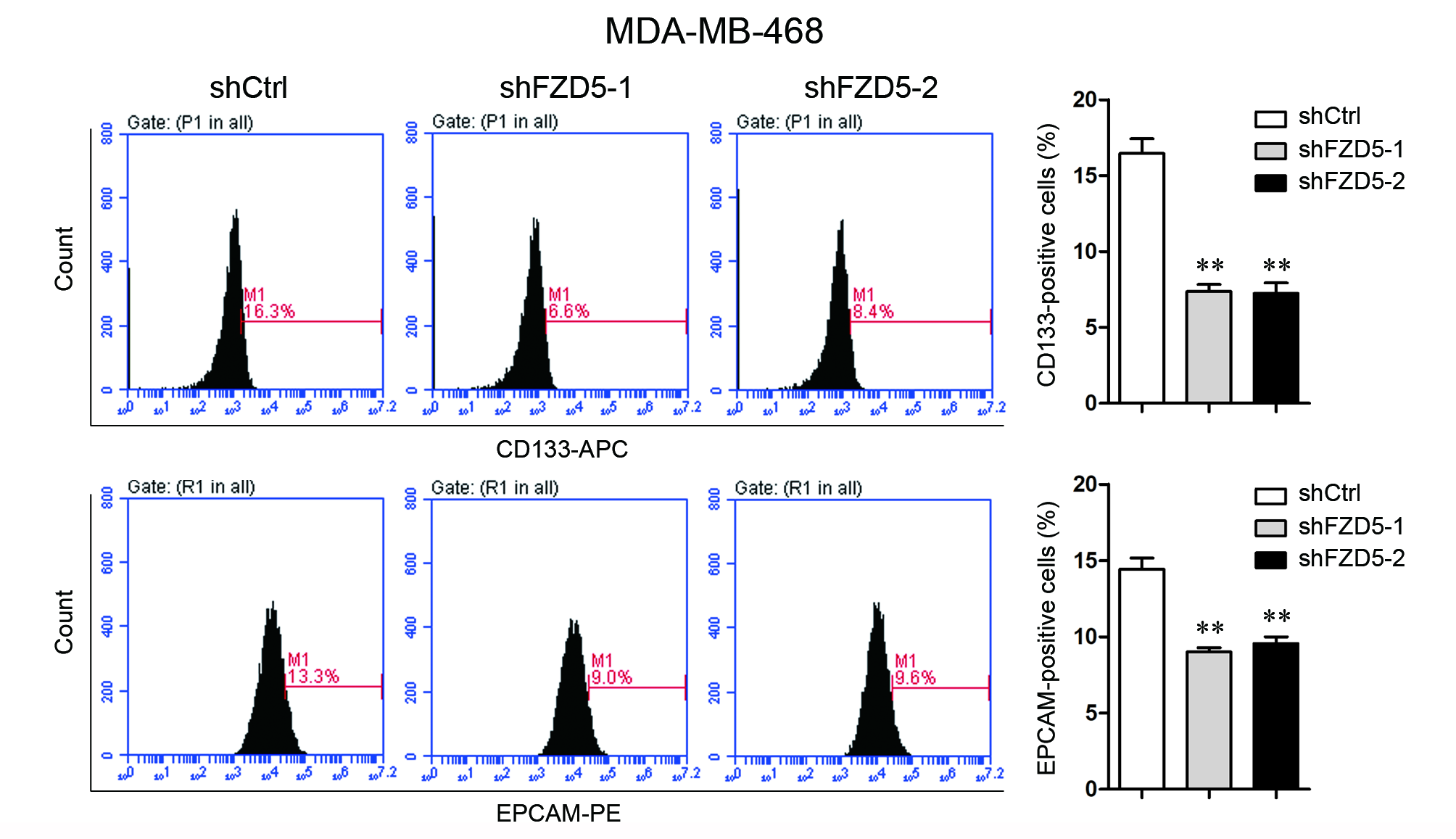

Supplement: Supplementary file 6 — Supplementary-Figure 6 [file 41419_2020_3282_MOESM6_ESM.tif]

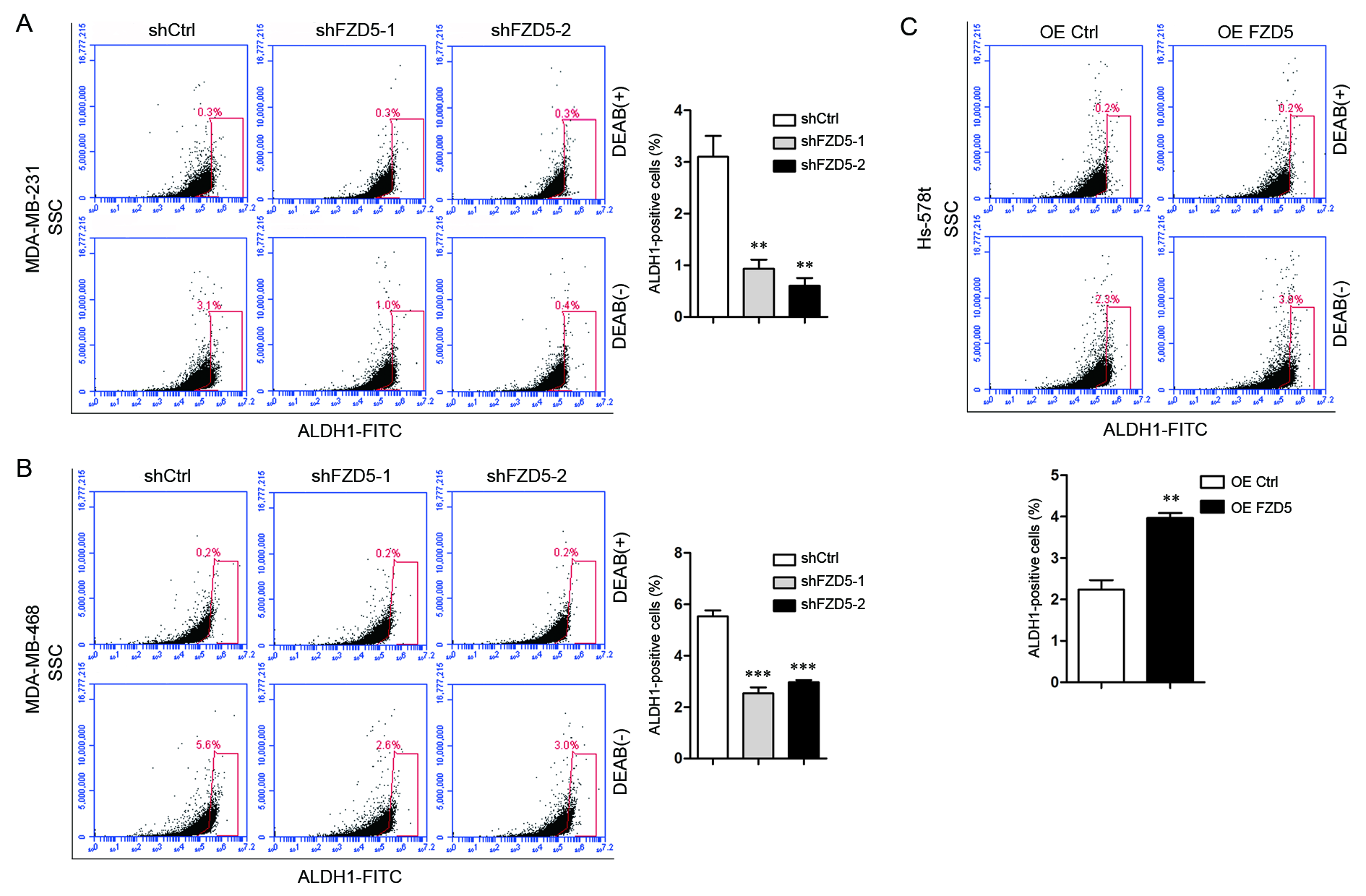

Supplement: Supplementary file 7 — Supplementary-Figure 7 [file 41419_2020_3282_MOESM7_ESM.tif]

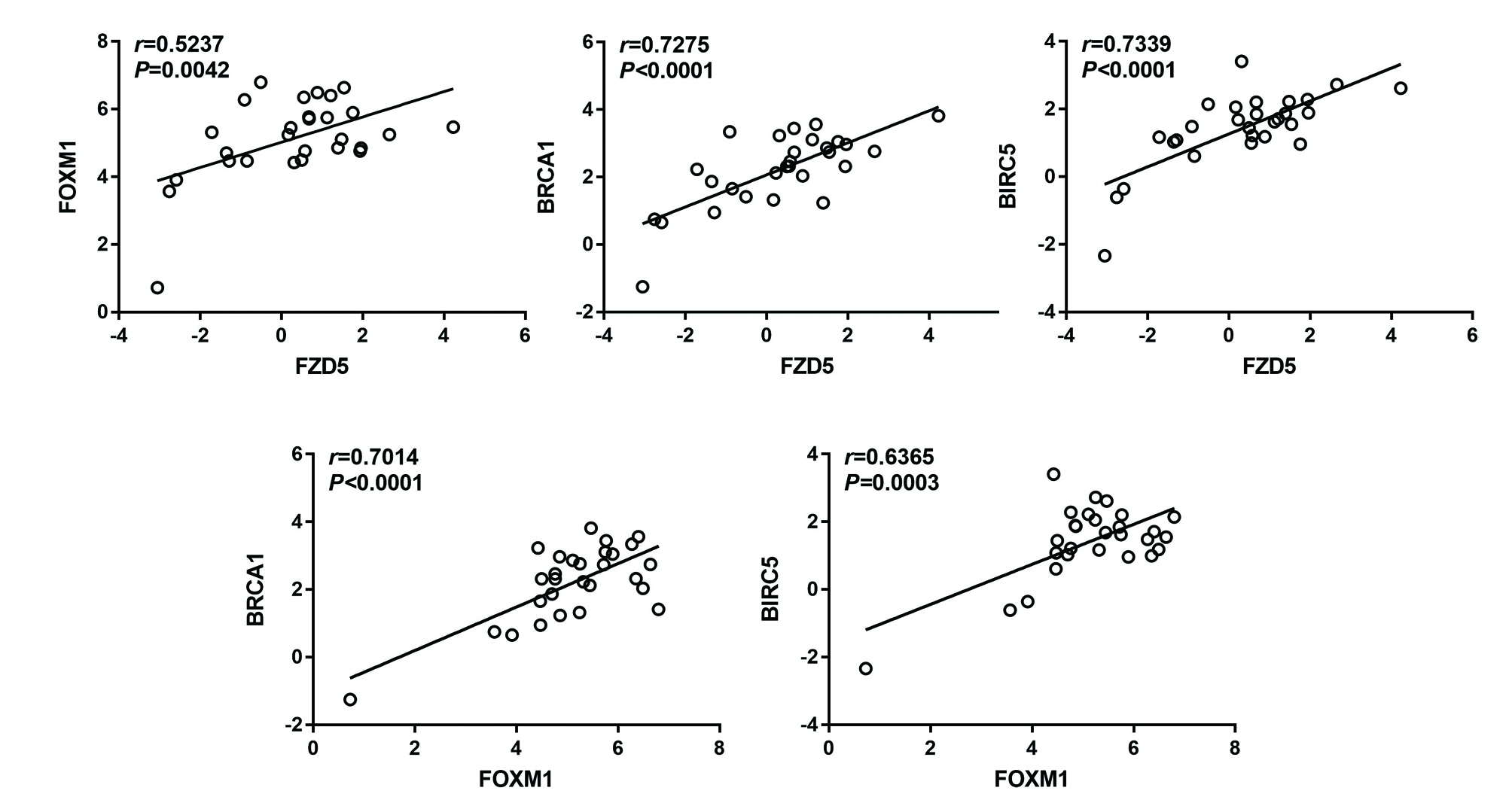

Supplement: Supplementary file 8 — Supplementary-Figure 8 [file 41419_2020_3282_MOESM8_ESM.tif]

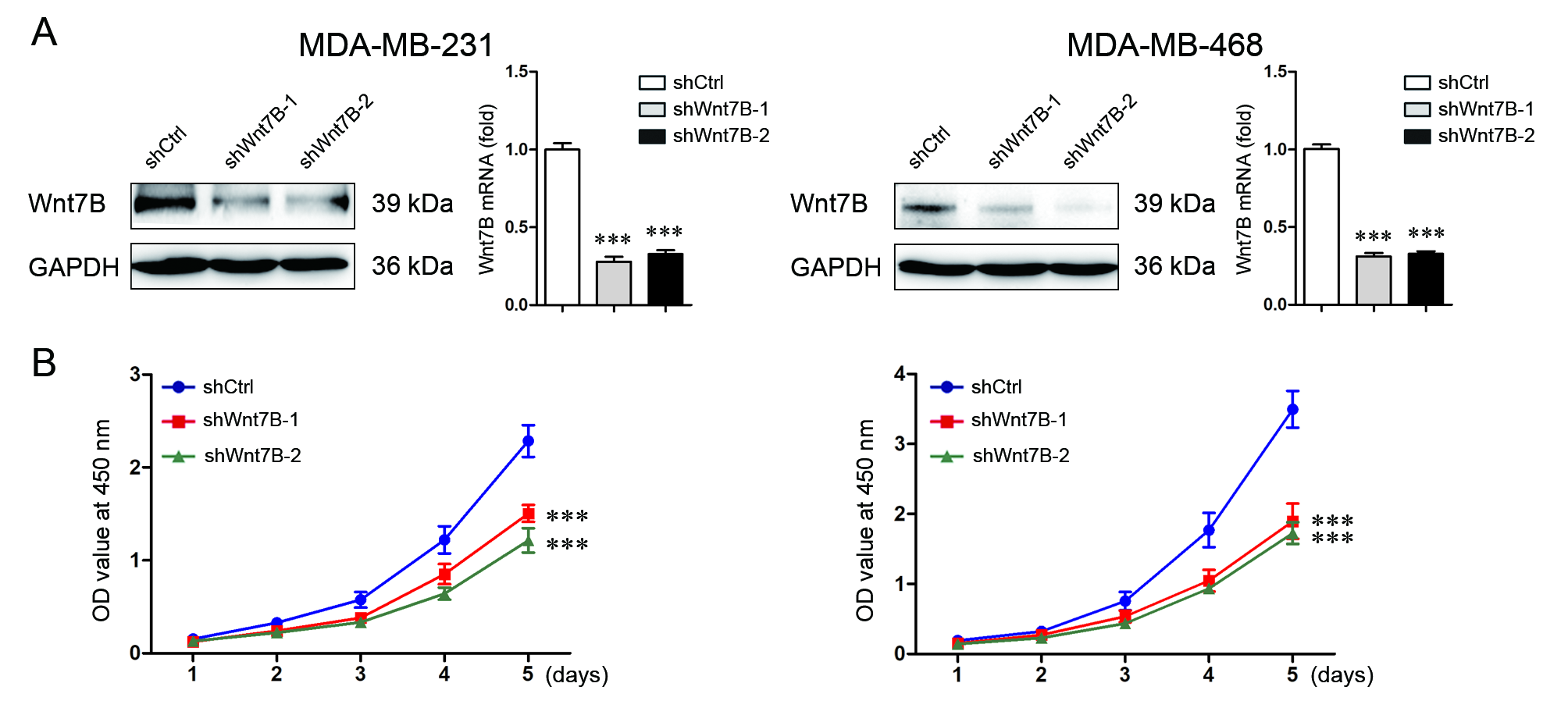

Supplement: Supplementary file 9 — Supplementary-Figure 9 [file 41419_2020_3282_MOESM9_ESM.tif]
