# Supplementary material for: FZD5 contributes to TNBC proliferation, DNA damage repair and stemness
Source: Cell Death Dis. 2020 Dec 12;11(12):1060. doi: 10.1038/s41419-020-03282-3 (PMC7733599; doi:10.1038/s41419-020-03282-3)
Supplement: Supplementary file 10 — Supplementary Figure Legends [file 41419_2020_3282_MOESM10_ESM.docx]

**Supplementary Figure legends**

**Supplementary Fig. 1: Gene Set Enrichment Analysis of TCGA database.** Go analysis of the gene pathways differentially expressed between FZD5-high and FZD5-low breast cancer samples in TCGA database was performed. 4 representative GSEA enrichment plots were shown.

**Supplementary Fig 2: Induction of cell growth by FZD5. A** FZD5 expression in MDA-MB-468 cells stably transfected with shCtrl or shFZD5-1/2 was detected by Western blot and Real-time PCR. **B** Viability of MDA-MB-468 cells was analyzed by CCK8. Mean±SD, n=3. ****P*<0.001, *vs* shCtrl. **C** Ki67 expression in xenograft tumors was detected by Immunohistochemistry. Scalebar: 25μm. Mean±SD, n=5. ***P*<0.01, *vs* shCtrl.

**Supplementary Fig 3: Promotion of G1/S transition and DNA replication by FZD5.** **A** Cell cycle of MDA-MB-468 cells was analyzed by Flowcytometry. **B** CDK2, Cyclin E2, Cyclin A2 and PCNA expression in MDA-MB-468 cells was detected by Western blot. C DNA replication of MDA-MB-468 cells was analyzed by EDU staining. Scalebar: 200μm. Mean±SD, n=3. ***P*<0.01, ****P*<0.001, vs shCtrl.

**Supplementary Fig. 4:** **Enhancement of DNA damage repair by FZD5. A** γ-H2AX expression in MDA-MB-468 cells was detected by Immunofluorescence 48 hours after treatment with ADR (300nM). Scalebar: 100μm. **B** EXO1, PLK4 and RFC4 expression in MDA-MB-468 cells was detected by Real-time PCR. Mean±SD, n=3. ***P*<0.01, ****P*<0.001, *vs* shCtrl.

**Supplementary Fig. 5:** **Enhancement of chemoresistance by FZD5.** **A** Death of MDA-MB-231 cells was detected by Flowcytometry 48 hours after treatment with Paclitaxel (500nM). **B** Death of Hs-578t cells was detected by Flowcytometry 48 hours after treatment with Paclitaxel (500nM).

**Supplementary Fig. 6: Maintenance of stem cell-like properties by FZD5.** Fractions of CD133-positive and EPCAM-positive MDA-MB-468 cells were detected by Flowcytometry. Mean±SD, n=3. ***P*<0.01, *vs* shCtrl.

**Supplementary Fig. 7: Maintenance of stem cell-like properties by FZD5.** Fractions of ALDH1-positive cells were detected by Flowcytometry. Mean±SD, n=3. ***P*<0.01, ****P*<0.001, *vs* shCtrl. **A** MDA-MB-231 cells. **B** MDA-MB-468 cells. **C** Hs-578t cells.

**Supplementary Fig. 8: Correlation analysis in CCLE database.** CCLE database was interrogated for FZD5, FOXM1, BRCA1 and BIRC5 expression. Correlation between two genes in 28 TNBC cell lines was analyzed by Pearson statistics.

**Supplementary Fig. 9: Induction of cell growth by Wnt7B. A** Wnt7B expression in MDA-MB-231 and MDA-MB-468 cells stably transfected with shCtrl or shWnt7B-1/2 was detected by Western blot and Real-time PCR. **B** Viability of MDA-MB-231 and MDA-MB-468 cells was analyzed by CCK8. Mean±SD, n=3. ****P*<0.001, *vs* shCtrl.
